# High prevalence of multidrug resistant *Enterobacteriaceae* among residents of long term care facilities in Amsterdam, the Netherlands

**DOI:** 10.1371/journal.pone.0222200

**Published:** 2019-09-12

**Authors:** Eline van Dulm, Aletta T. R. Tholen, Annika Pettersson, Martijn S. van Rooijen, Ina Willemsen, Peter Molenaar, Marjolein Damen, Paul Gruteke, Paul Oostvogel, Ed J. Kuijper, Cees M. P. M. Hertogh, Christina M. J. E. Vandenbroucke-Grauls, Maarten Scholing

**Affiliations:** 1 Department of Infectious Diseases, Public Health Service Amsterdam, Amsterdam, the Netherlands; 2 Centre for Zoonoses and Environmental Microbiology, National Institute for Public Health and the Environment (RIVM), Bilthoven, the Netherlands; 3 Department of Medical Microbiology and Infection Control, Amsterdam UMC, Vrije Universiteit Amsterdam, Amsterdam, the Netherlands; 4 Department of Medical Microbiology and Infection Control, Amphia Hospital, Breda, the Netherlands; 5 National Coordination Centre for Communicable Disease Control, National Institute for Public Health and the Environment (RIVM), Bilthoven, the Netherlands; 6 Department of Medical Microbiology, Maasstad General Hospital, Rotterdam, the Netherlands; 7 Department of Medical Microbiology, OLVG Lab BV, Amsterdam, the Netherlands; 8 Department of Medical Microbiology, Leiden University Medical Center, Leiden, the Netherlands; 9 Department of General Practice & Elderly Care Medicine, Amsterdam UMC, Vrije Universiteit Amsterdam, Amsterdam, the Netherlands; Wadsworth Center, UNITED STATES

## Abstract

**Introduction:**

The aim of this study was to determine the rate of asymptomatic carriage and spread of multidrug-resistant micro-organisms (MDRO) and to identify risk factors for extended spectrum beta-lactamase-producing *Enterobacteriaceae* (ESBL-E) carriage in 12 long term care facilities (LTCFs) in Amsterdam, the Netherlands.

**Materials and methods:**

From November 2014 to august 2015, feces and nasal swabs from residents from LTCFs in Amsterdam, the Netherlands were collected and analyzed for presence of multidrug-resistant Gram-negative bacteria (MDRGN), including ESBL-E, carbapenemase-producing *Enterobacteriaceae* (CPE), colistin-resistant *Enterobacteriaceae* and methicillin-resistant *Staphylococcus aureus* (MRSA) and vancomycin-resistant enterococci (VRE). Logistic regression analysis was performed to assess associations between variables and ESBL-carriage.

**Results:**

In total, 385 residents from 12 LTCFs (range 15–48 residents per LTCF) were enrolled. The prevalence of carriage of MDRGN was 18.2% (range among LTCFs 0–47%) and the prevalence of ESBL-E alone was 14.5% (range among LTCFs: 0–34%). Of 63 MDRGN positive residents, 50 (79%) were ESBL-E positive of which 43 (86%) produced CTX-M. Among 44 residents with ESBL-E positive fecal samples of whom data on contact precautions were available at the time of sampling, only 9 (20%) were already known as ESBL-E carriers. The prevalence for carriage of MRSA was 0.8% (range per LTCF: 0–7%) and VRE 0%. One CPE colonized resident was found. All fecal samples tested negative for presence of plasmid mediated resistance for colistin (MCR-1). Typing of isolates by Amplified Fragment Length Polymorphism (AFLP) showed five MDRGN clusters, of which one was found in multiple LTCFs and four were found in single LTCFs, suggesting transmission within and between LTCFs. In multivariate analysis only the presence of MDRO in the preceding year remained a risk factor for ESBL-E carriage.

**Conclusions:**

The ESBL-carriage rate of residents in LTCFs is nearly two times higher than in the general population but varies considerably among LTCFs in Amsterdam, whereas carriage of MRSA and VRE is low. The majority (80%) of ESBL-E positive residents had not been detected by routine culture of clinical specimens at time of sampling. Current infection control practices in LTCFs in Amsterdam do not prevent transmission. Both improvement of basic hygiene, and funding for laboratory screening, should allow LTCFs in Amsterdam to develop standards of care to prevent transmission of ESBL-E.

## Introduction

Antimicrobial resistance has been identified as a key public health challenge [1]. Amongst the multidrug-resistant micro-organisms (MDRO) are extended spectrum beta-lactamase-producing *Enterobacteriaceae* (ESBL-E), carbapenemase-producing *Enterobacteriaceae* (CPE), vancomycin-resistant enterococci (VRE), and methicillin-resistant *Staphylococcus aureus* (MRSA). The Netherlands is a country with low antibiotic use in humans and is among to the countries with the lowest antibiotic resistance rates in clinical isolates in Europe [2].

Dutch national guidelines for contact precautions for carriers of MDRO (other than MRSA) in Long Term Care Facilities (LTCFs) were published late 2014 [3]. In addition to European guidelines for the management of infection control precautions of multidrug-resistant Gram-negative bacteria (MDRGN) in hospitals [4], the Dutch national guidelines also define co-resistance to fluoroquinolones and aminoglycosides in *Enterobacteriaceae* as multidrug resistance. *Pseudomonas aeruginosa* is considered MDRGN when resistance for three out of five of the following antibiotics is detected: carbapenems, aminoglycosides, fluorochinolones, ceftazidim, piperacillin[3].

Previous studies in Amsterdam (2010–2011) showed a prevalence of ESBL-E carriage of 10.6% (95% CI: 9.7–11.5) and 8.6% (95% CI: 7.3–10.0) in patients attending their general practitioner with gastrointestinal symptoms and in the general population, respectively [5, 6]. A point prevalence study among 200 patients screened upon admission in a large general hospital in Amsterdam in 2014 [7], showed a MDRGN prevalence of 10.5%, of which 76% was identified as ESBL-E.

Outbreaks of MDRGN are rarely detected and only incidentally reported in Dutch LTCFs [8]. Point prevalence studies in Dutch LTCFS have shown a large variation in MDRO carriage rates, ranging from 4% to 21% [8–12]. The role of LTCFs in the transmission of MDRO within the Dutch healthcare network and interventions needed to prevent transmission of MDRO in LTCFs are still under debate [13, 14]. Our aim was to study the prevalence, risk factors and molecular epidemiology of carriage of MDRO among residents of LTCFs in Amsterdam.

## Materials and methods

### Setting and data collection

For this cross-sectional study we made a selection of LTCFs in Amsterdam that provided assisted-living and intensive nursing and harbored at least 50 residents. LTCFs with different types of nursing wards (psychogeriatric, somatic, rehabilitation, or a combination of these wards) were included to obtain an equal number of patients of each type of ward. Resident-related risk factors for carriage of MDRO were assessed by a questionnaire that was completed by LTCF nursing staff. Institutional risk factors were assessed through a questionnaire that was completed by the LTCF management staff and during a site visit at participating LTCF wards by an expert in infection control. Risk factors were scored, using scoring lists adapted from a previously validated infection risk scan (IRIS) [12].

### Sample collection

Nasal swabs (Copan eMRSA^™^, Brescia, Italy) and feces (COPAN FecalSwab^™^, Brescia, Italy) were collected from each participating resident by local nursing staff.

### MDRGN definition

MDRGN were defined as used by the Dutch national guidelines. *Enterobacteriaceae* were considered MDRGN when they were ESBL or carbapenemase-producing or if they harbored a co-resistance

### Laboratory detection

After overnight incubation (37°C), nasal swabs were cultured on chromID™ MRSA agar (bioMerieux, Marcy l’Etoile, France). Feces was cultured on chromID™ MRSA agar after overnight incubation in nutrient broth no.2 + 6% NaCl (Media Products, Groningen, the Netherlands). Feces was additionally screened for 1) multidrug-resistant Gram-negative organisms using overnight incubation of an amoxicillin (16mg/L) containing BHI broth (Media Products) subcultured to MacConkey agar plates (Media Products) with cefotaxime (5ug) and ceftazidim (10 ug) neo-sensitabs (Rosco Diagnostica, Taastrup, Denmark) and MacConkey agar plates containing 16 ug/L gentamicin with a ciprofloxacin neo-sensitab (10ug) and 2) VRE using overnight incubation of an antibiotic free Enterococcosel^TM^ enrichment broth (Becton Dickinson, Utrecht, Netherlands) and chromID™ VRE (bioMerieux) agar plates. Identification and antimicrobial susceptibility testing (N200 card) of isolates was performed by standard methods and phenotypic confirmation of ESBL by E-test in accordance with EUCAST [15] and Dutch national guidelines [16]. Confirmation and genotyping of MRSA and CPE was performed by the Dutch reference laboratory at the National Institute for Public Health and the Environment (RIVM). Amplified fragment length polymorphism (AFLP) was performed on all available multidrug-resistant *Enterobacteriaceae* isolates as described in the S1 File. Isolates were considered indistinguishable (representing a cluster) when the band patterns were >90% identical. For phylogenetic typing, a selection of *Escherichia coli* isolates were further analyzed by phylogroup-defining PCR [17]. Group B2 *E*. *coli* were further characterized by O25:ST131-specific PCR [18]. All phenotypically ESBL-positive isolates were tested for the presence of CTX-M, SHV and TEM ESBL resistance genes by PCR as previously described [19, 20]. The CTX-M, SHV and TEM ESBL resistance genes were additionally typed by sequencing. Sequencing was performed as described in the S1 File. Primer sequences are listed in S1 Table. Consensus sequences were uploaded at The Comprehensive Antibiotic Resistance Database BLAST service for typing (Jia et al., at http://arpcard.mcmaster.ca) [21]. The MCR-1 PCR was performed by the Department of Medical Microbiology of Leiden University Medical Centre according to methods described previously by Nijhuis et al. [22] and Terveer et al. [23]. All laboratory detection methods are described in the S1 File.

### Statistical analysis

Associations between variables and ESBL carriage were assessed by univariable logistic regression analysis. All variables with an associated p<0.25 in univariable analyses were included in a multivariable model, with the exception of type of room and use of contact precautions at the time of sampling (since these factors might be a consequence of previously detected ESBL-carriage). A backwards-stepwise procedure was performed by sequentially removing any variable with a p-value >0.05 in order to obtain a final multivariable model. Results are presented as odds ratios (OR) with corresponding 95% confidence intervals (CI). Confidence intervals that did not contain 1 were considered statistically significant. All statistical analyses were performed with SPSS version 21.0 (SPSS Inc., Chicago, IL, USA).

### Ethical considerations

This study was reviewed and approved by the Medical Ethical Committee of the VU Medical Center Amsterdam (protocol ID NL50241.018.14). The study was judged to be beyond the scope of the Medical Research Involving Human Subjects Act (in Dutch, Wet Medisch-wetenschappelijk Onderzoek met Mensen [WMO]), and a waiver of written informed consent was obtained. Patients who participated in the study provided verbal informed consent for use of demographic, clinical, and culture data.

## Results

### Participating LTCFs and residents

Twenty-four Amsterdam LTCFs were approached of whom ten participated in this study. Because of a lower response rate than expected we additionally approached one LTCF in Zaandam (LTCF L, 15km from Amsterdam) and one LTCF post acute care ward located in a large teaching hospital (<50 residents, LTCF K). The main reason for non-participation was the expected workload of sampling. Characteristics of the participating LTCFs are listed in Table 1. From November 2014 to August 2015, 385 residents from 12 LTCFs (range 15–48 residents per LTCF, 1730 residents in total) were enrolled. For 30 residents sample collection was either not or inadequately performed and they were excluded from further analysis. For another 36 residents the questionnaire was missing. For 310 residents both a fecal swab and questionnaire were available for analysis for MDRO colonization. Key participant characteristics of residents with both a fecal swab and questionnaire available for analysis are summarized in Table 2.

**Table 1:** Characteristics and MDRGN prevalence of 12 participating LTCFs.

LTCF	No. of residents	No. of samples	ESBL+ residents		Total MDRGN^#^		MDRGN cluster analysis by AFLP
			N	%	N	%	
**A**	125*	34	6	17.6%	7	20.6%	Cluster 1 (5 residents), Cluster 5
**B**	130	34	6	17.6%	7	20.6%	Cluster 1 (2 residents), Cluster 4
**C**	193	42	2	4.8%	5	11.9%	
**D**	189	17	0	0.0%	0	0.0%	
**E**	108	17	0	0.0%	0	0.0%	
**F**	144	32	11	34.4%	15	46.9%	Cluster 1 (4 residents), Cluster 2, Cluster 3
**G**	110	33	6	18.2%	7	21.2%	
**H**	199	39	6	15.4%	8	20.5%	Cluster 1 (1 resident)
**I**	144	32	9	28.1%	9	28.1%	
**J**	96	18	1	5.6%	2	11.1%	
**K**	20	13	3	23.1%	3	23.1%	
**L**	272	35	0	0.0%	0	0.0%	
**Total**	**1,730**	**346**	**50**	**14.5%**	**63**	**18.2%**	

# Includes ESBL-E, Carbapenemase-producing *Enterobacteriaceae*, Multidrug-resistant *P*. *aeruginosa* and aminoglycoside-fluoroquinolones co-resistant *Enterobacteriaceae*

* Estimated number based on historical data

Abbreviations: MDRGN = Multidrug-resistant Gram-negative bacteria; LTCF = Long term care facility; No. = number; ESBL-E = Extended Spectrum Beta Lactamase-producing *Enterobacteriaceae*; AFLP = Amplified Fragment Length Polymorphism

**Table 2:** Demographics and clinical characteristics of participating residents. Cases are ESBL-E carriers. Univariable associations of demographic and clinical characteristics with ESBL carriage of LTCF participants with both a fecal swab and questionnaire available for analysis (N = 310).

Variable	Cases*/Total		OR	95%CI	p-value
	N	%			
**Sex**					.590
Female	30/199	15.1%	Ref		
Male	14/109	12.8%	0.83	0.42–1.64	
**Age**					.168
<70 years	2/39	5.1%	Ref		
70–79 years	11/61	18.0%	4.07	0.85–19.47	
80–89 years	22/129	17.1%	3.80	0.85–16.96	
≥90 years	9/73	12.3%	2.60	0.53–12.69	
**Nursing indication**					.689
Psychogeriatric	14/108	13.0%	Ref		
Somatic	19/137	13.9%	1.08	0.51–2.27	
Rehabilitation	11/62	17.7%	1.45	0.61–3.42	
**Antimicrobial use in previous 30 days**					.899
No	39/273	14.3%	Ref		
Yes	5/37	13.5%	0.94	0.34–2.55	
**Current antimicrobial use**					.888
No	43/302	14.2%	Ref		
Yes	1/8	12.5%	0.86	0.10–7.17	
**Hospitalization in previous 90 days**					.449
No	33/218	15.1%	Ref		
Yes	9/77	11.7%	0.74	0.34–1.63	
**MDRO detected in previous year**					< .001
No	35/289	12.1%	Ref		
Yes	9/15	60.0%	10.89	3.65–32.43	
**Type of room**					
Single person	32/201	15.9%	#	#	
Multiple person	9/88	10.2%	#	#	
**Contact precautions at time of sampling**					
No	35/293	12.0%	#	#	
Yes	9/14	64.3%	#	#	
**Length of stay**					.401
0–10 weeks	13/73	17.8%	Ref		
11–64 weeks	7/74	9.5%	0.48	0.18–1.29	
65–161 weeks	10/73	13.7%	0.73	0.30–1.80	
162–670 weeks	13/73	17.8%	1.00	0.43–2.33	
**Decubitus wounds**					.796
No	41/285	14.4%	Ref		
Yes	3/24	12.5%	0.85	0.24–2.98	
**Other wounds**					.534
No	39/279	14.0%	Ref		
Yes	5/27	18.5%	1.40	0.50–3.91	
**Pneumonia in medical history**					.508
No	34/251	13.6%	Ref		
Yes	10/59	16.9%	1.30	0.60–2.81	
**Comorbidities**					
**Diabetes**					.097
No	29/236	12.3%	Ref		
Yes	15/74	20.3%	1.81	0.92–3.61	
**COPD**					.050
No	34/270	12.6%	Ref		
Yes	10/40	25.0%	2.31	1.04–5.15	
**Vascular disorder**					.819
No	21/143	14.7%	Ref		
Yes	23/167	13.8%	0.93	0.49–1.76	
**Renal impairment**					.893
No	33/235	14.0%	Ref		
Yes	11/75	14.7%	1.05	0.50–2.20	
**IBD**					
No	44/305	14.4%	-	-	
Yes	0/5	0.0%	-	-	
**Other**					
No	43/307	14.0%	Ref		
Yes	1/3	33.3%	3.07	0.27–34.59	.401
**Current infections**					
**Sepsis/bacteremia**					
No	44/310	14.2%	-	-	
Yes	0/0	-	-	-	
**Urinary tract infection**					.901
No	42/297	14.1%	Ref		
Yes	2/13	15.4%	1.10	0.24–5.16	
**Upper respiratory tract infection**					.153
No	42/305	13.8%	Ref		
Yes	2/5	40.0%	4.17	0.68–25.73	
**Lower respiratory tract infection**					.994
No	43/303	14.2%	Ref		
Yes	1/7	14.3%	1.01	0.12–8.58	
**Gastro-intestinal tract infection**					
No	44/307	14.3%	-	-	
Yes	0/3	0.0%	-	-	
**Skin infection**					.994
No	43/303	14.2%	Ref		
Yes	1/7	14.3%	1.01	0.12–8.58	
**Medical devices**					
**Urinary catheter**					.361
No	42/286	14.7%	Ref		
Yes	2/24	8.3%	0.53	0.12–2.33	
**Suprabubic catheter**					
No	44/303	14.5%	-	-	
Yes	0/7	0.0%	-	-	
**PEG tube**					.721
No	43/305	14.1%	Ref		
Yes	1/5	20.0%	1.52	0.17–13.95	
**Vacuum therapy**					
No	43/294	14.6%	-	-	
Yes	0/0	-	-	-	
**Intravascular catheter**					
No	44/309	14.2%	-	-	
Yes	0/1	0.0%	-	-	
**Incontinence**					
**Urine**					.672
No	19/143	13.3%	Ref		
Yes	25/167	15.0%	1.14	0.60–2.19	
**Feces**					.926
No	22/157	14.0%	Ref		
Yes	22/153	14.4%	1.03	0.54–1.95	

* Cases are defined as carriers of ESBL

# Not estimated since contact measures at time of sampling and staying in a single vs. multiple person room might be a consequence of known ESBL-E carriage

Missings: sex 2; age 8; nursing indication 3; decubitis wounds 1; other wounds 4; hospitalization in previous 90 days 15; MDRO detected in previous year 6; type of room 21; ICP at time of sampling 3; length of stay 17; vacuum therapy 16

Abbreviations: CI = Confidence interval; OR = Odds ratio; IQR = Inter quartile range; COPD = Chronic Obstructive Pulmonary Disease; IBD = Inflammatory Bowel Disease; PEG = Percutaneous Endogastric; MDRO = Multidrug-resistant micro-organisms; ESBL = Extended Spectrum Beta Lactamase

### Prevalence of carriage of MDRO

The prevalence of carriage of MDRGN was 18.2% (range among LTCFs 0–47%) and the prevalence of ESBL-E alone was 14.5% (range among LTCFs: 0–34%) (Table 1). The prevalence of carriage of MRSA was 0.8% (range per LTCF: 0–7%) and of VRE 0%. The three carriers of MRSA resided in three different LTCFs and did not carry MDRGN. In total, 71 unique MDRGN isolates were cultured from 63 residents; 53/71 (75%) isolates from 50 residents phenotypically produced ESBL, of which 39 (74%) were identified as *E*. *coli*, 12 (23%) as *Klebsiella pneumoniae* and two as *Enterobacter cloacae* and *Citrobacter freundii*. Thirteen ESBL-producing isolates were co-resistant to fluoroquinolones and aminoglycosides and one *K*. *pneumoniae* isolate also produced New Delhi Metallo-beta-lactamase-1 (NDM). The prevalence of ESBL-E carriage among LTCF residents was 14.5% (95% CI: 10.8–18.2). Of the remaining 18 non-ESBL isolates, 17 *Enterobacteriaceae* were resistant to the combination of aminoglycosides and fluoroquinolones and one isolate identified as *P*. *aeruginosa* additionally resistant to piperacillin. All 346 fecal samples were negative for presence of the MCR-1 gene.

### Molecular characterization and ESBL typing

A total of 7/71 (10%) MDRGN isolates had not been stored and could not be retrieved from the small quantities of original sample kept by -80 degrees Celsius. The missing isolates have a similar distribution of species identification, resistance pattern and LTCF location as the selection used for molecular analysis.

**Fig 1:**
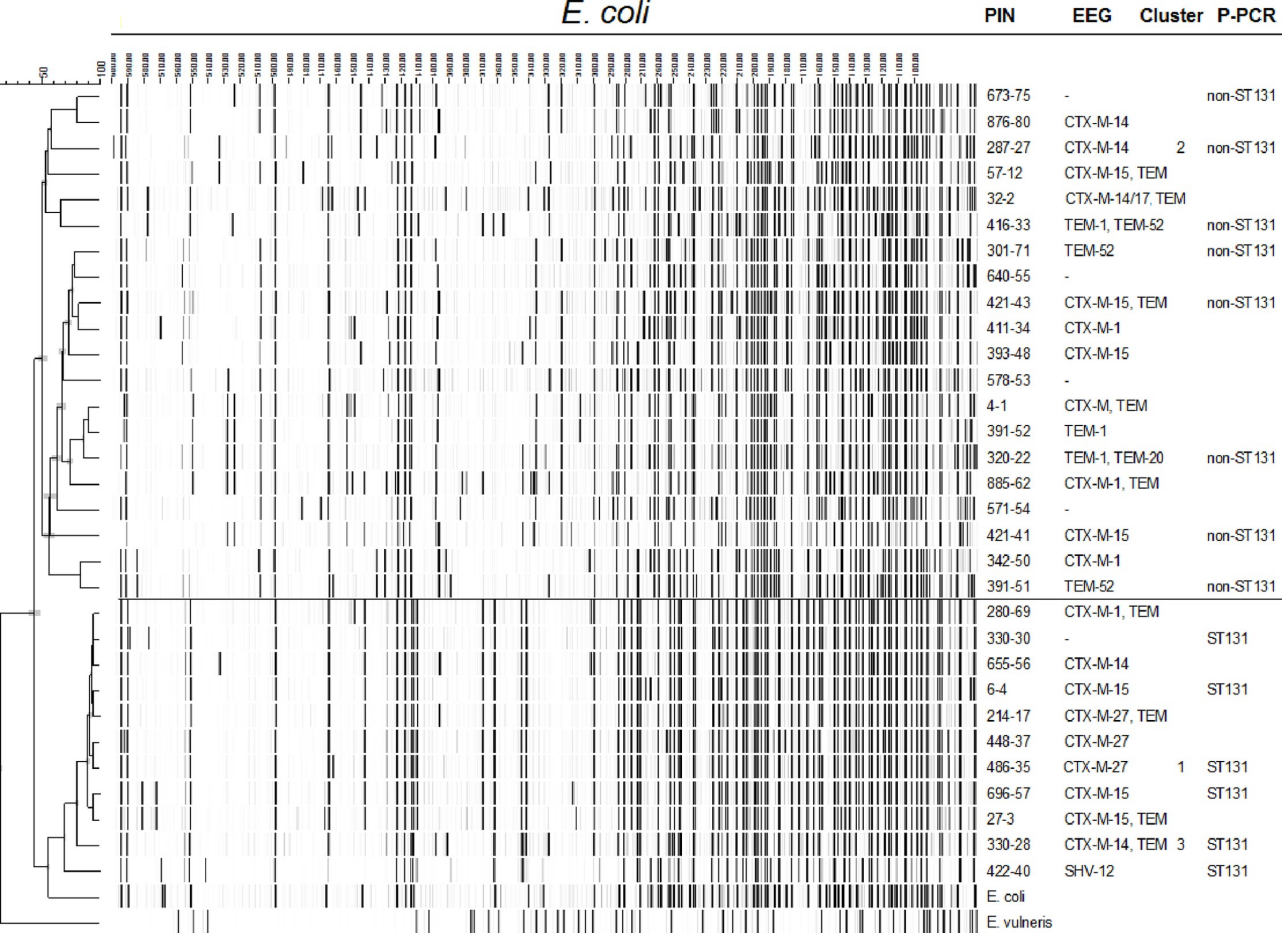
AFLP-results of all *E*. *Coli* isolates with one representative isolate per cluster. Abbreviations: PIN = Patient Identification Number; EEG = ESBL Encoding Gene; P-PCR = Phylogroup defining Polymerase Chain Reaction.

**Fig 2:**
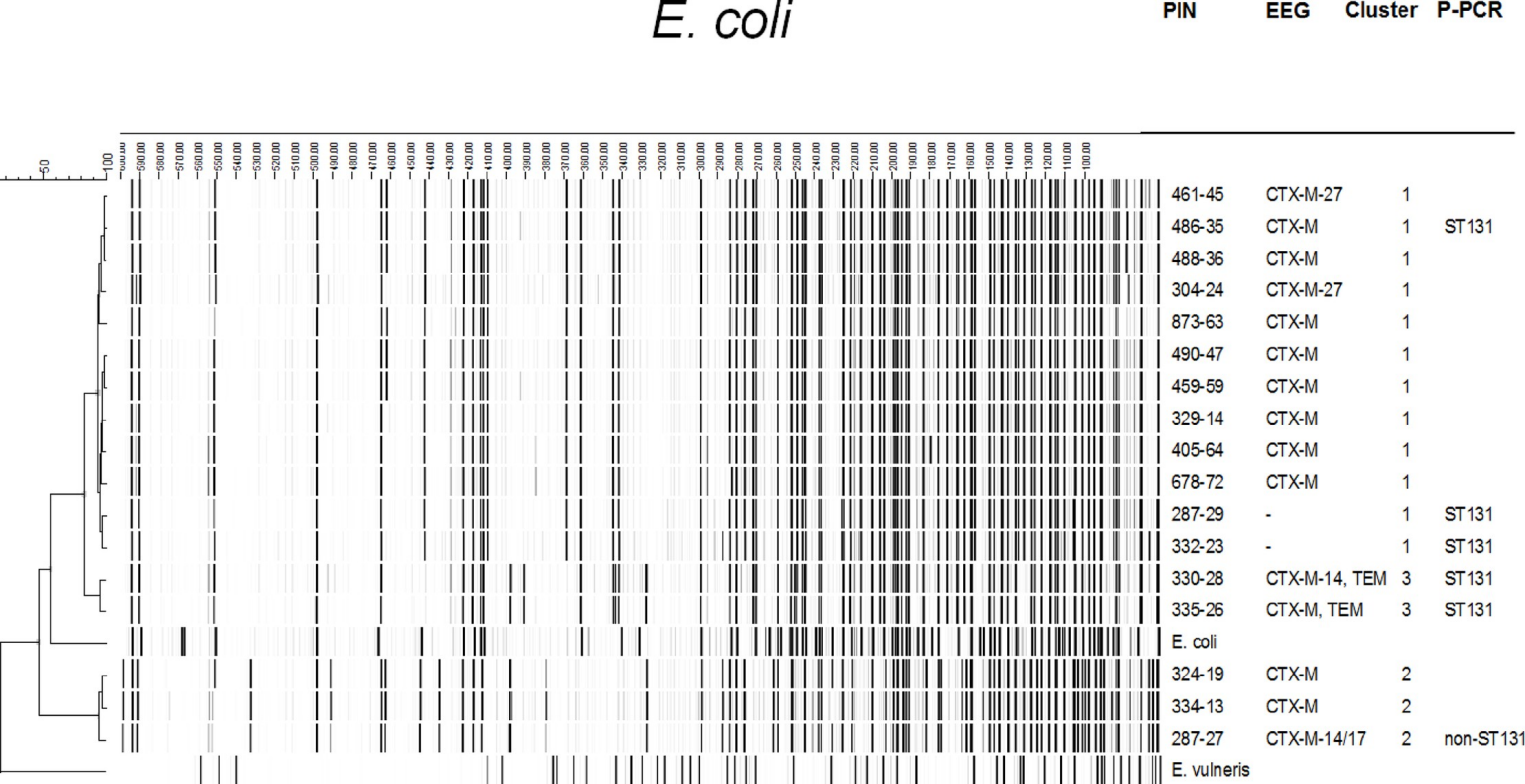
*E*. *Coli* isolates from clusters 1–3. Abbreviations: PIN = Patient Identification Number; EEG = ESBL Encoding Gene; P-PCR = Phylogroup defining Polymerase Chain Reaction.

The presence of genes encoding ESBL was confirmed in all phenotypically ESBL-producing isolates except for one isolate which had a TEM-1 gene only (no ESBL). However, another *E*. *coli* isolate of the same resident with a different AFLP-result was genotypically confirmed as ESBL. The ESBL-genes most frequently detected were CTX-M-15 (16/51, 31%) and CTX-M-27 (12/51, 24%) (Table 3). In total, 5 clusters varying in size from 2 to 12 strains, were detected in 4 LTCFs by phylogenetic analysis of AFLP-results (Table 1). Fig 1 depicts AFLP-results of all *E*. *coli* isolates with one representative isolate per cluster. All *E*. *coli* isolates from clusters 1–3 are depicted in Fig 2. A total of 22/63 (35%) MDRGN carriers from our study could be clustered with at least one other MDRGN carrier.

Phylogenetic typing was performed on a selection of 19 *E*. *coli* isolates, dividing the phylogenetic tree in half with 22/45 (49%) non-ST131 *E*. *coli* isolates and 23/45 (51%) ST131 *E*. *coli* isolates (Fig 1). Isolates from clusters 1 and 3 belong to the ST131 genotype.

**Table 3:** ESBL-encoding genes (1 sequence per cluster, see Methods).

ESBL family	ESBL gene/type	N
**CTX-M-1 family**	*bla*_CTX-M-15_	16*
	*bla*_CTX-M-1_	4
**CTX-M-9 family**	*bla*_CTX-M-14_ *bla*_CTX-M-14 /17__$_	7 1
	*bla*_CTX-M-9_	2
	*bla*_CTX-M-27_	12
**CTX-M**^**#**^	*bla*_CTX-M_	1
**TEM and SHV**^**+**^	*bla*_TEM-52_	3
	*bla*_TEM-20_	1
	*bla*_SHV-2_	1
	*bla*_SHV-12_	2
**Total**		**50**

* One isolate also encoded New Delhi Metallo-beta-lactamase-1

$ No discrimination between CTX-M-14 and CTX-M-17. One strain was phenotypically ESBL, but no ESBL gene could be detected

# Exact subtype of one CTX-M gene remained unresolved by sequencing

+ Possibly, there were more TEM or SHV ESBL genes present. TEM or SHV was not sequenced from CTX-M-positive strains

Abbreviations: N = number; ESBL = Extended Spectrum Beta Lactamase

### Risk factors for carriage of ESBL

For 310 of 385 residents both results from fecal sample cultures and from questionnaire were available; these were used for analysis of resident-related risk factors for carriage of ESBL-E. Among 44 residents with ESBL-E positive fecal samples of whom data on contact precautions were available at the time of sampling, only 9 (20%) were already known as ESBL-E carriers. In the univariable logistic regression analysis the following risk factors (p<0.25) were associated with ESBL-carriage: age, MDRO carriage in the preceding year, diabetes mellitus, COPD and having a current upper respiratory tract infection (Table 2). In the multivariable logistic regression analyses only the presence of a MDRO in the preceding year remained a risk factor for ESBL-carriage (OR 10.9, 95%CI: 3.7–32.4).

## Discussion

The present study showed that nearly one in five residents carried MDRGN in LTCFs in Amsterdam. Phylogenetic analysis showed five clusters of isolates in four LTCFs, suggesting transmission of ESBL-E within and between LTCFs. The large majority of MDRGN were ESBL-E, with a prevalence of carriage of nearly one in seven residents. The prevalence of MRSA was less than 1%, while no carriers of VRE were found.

Our study suggests a higher prevalence of ESBL-E carriage in nursing home residents than in the general population in the Amsterdam area. The majority of these carriers was only detected during the prevalence survey, hence, most carriers remain undetected.

Verhoef et al [9] found an overall prevalence of ESBL-resistance genes of 4.2% in *E*. *coli* isolates isolated from urine samples in 107 Dutch LTCFs in 2012–2014. Only one LTCF from Amsterdam participated in that survey, and no cases of ESBL-E were detected among its residents. This prevalence is likely to be an underestimation because Verhoef only focused on ESBL-producing *E*. *coli* in urine samples, and not on gastrointestinal carriage of ESBL-E. LTCFs in the Amsterdam area are underrepresented in national surveillance studies such as SNIV (surveillance network in LTCFs), hampering actual insight and control plans for MDRO. However, healthcare inspectorate reports suggest that quality and safety of care in LTCFs in Amsterdam are compromised more often compared to acute care facilities [9, 24].

Preliminary results of a recent national surveillance point-prevalence study for intestinal carriage of resistant bacteria show an ESBL-E prevalence of 9.5% (range 0–22%) in eight nursing homes where feces samples were collected from 337/448 (75%) of residents [10]. In other Dutch studies, fecal ESBL-carriage was demonstrated in 70/643 (10.9%) nursing home residents [12] and in 50/579 (8.6%) residents of nursing homes screened upon hospital admission compared to 61/772 (7.9%) elderly who still lived in their own homes [11]. A study performed in region Leiden revealed fecal ESBL-carriage of 11% (E.M. Terveer and E.J. Kuijper, manuscript submitted).

The ESBL-E prevalence in our study was significantly higher than that of nearly 9% found in the general population in Amsterdam in 2011 [6]. In that study, age was not associated with a higher risk of ESBL-E carriage. Although the prevalence of ESBL-E may have increased in the general population since 2011, our study indicates that LTCFs in Amsterdam may represent a potential reservoir for MDRO in the healthcare network.

The majority of ESBL-E carriers was not detected by routine culture of clinical specimens and were only detected during the prevalence survey. The high proportion of ESBL-E carriers that were additionally detected in our study, may be explained by the restrictive diagnostic policy in LTCFs, and the absence of surveillance. Applying additional contact precautions only to the few known carriers of ESBL-E will very likely result in on-going transmission among residents and to other healthcare institutions. The current infection control policy, which does not include surveillance or regular screening, is likely to be ineffective.

The ESBL-E carriage prevalence ranged from 0% to 34% between participating LTCFs in our study. In a previous survey of a single LTCF in the South of the Netherlands, ESBL-E carriage rates varied substantially between wards, between 0% and 47% [8]. This means that the outcome of a single survey is highly dependent on the selection of wards in the LTCF. This also indicates that good quality prognostic determinants of ESBL-E transmission in LTCFs are needed.

The distribution of ESBL-encoding genes in our study is similar to that in the general population of Amsterdam [6] with the exception of CTX-M-27, which was more prevalent in nursing homes. This, however may be related to the presence of a cluster of isolates with this gene (cluster 1). While nearly 16% of ESBL-E in the general population of Amsterdam belong to the ST131 MLST genotype, in LTCFs, nearly 50% of ESBL-E belong to this easily expanding, more virulent and better persisting genotype [25, 26]. The only cluster of isolates that extended over more than one LTCF in our study belonged to ST131. The overrepresentation of ST131 in LTCFs could be due to clonal expansion since the study performed in the general population, or may be due to a higher transmission rate of ESBL-E (or exposure to a common source) in LTCFs. ST131 clone is associated with community-acquired infections and older age and is frequently observed in nursing homes throughout Europe [27].

In our study, one third of MDRGN isolates could be clustered with at least one other MDRGN isolate, suggesting a high transmission rate of MDRGN. A similar high rate (54%) was found in two geriatric rehabilitation wards in Israel [28]. In a recent study, Kluytmans-van den Berg et al. analyzed 2005 ESBL-E isolates from 690 ward-based prevalence surveys performed in 14 Dutch hospitals over a period of three years. With core genome Multilocus Sequence Typing (cgMLST) they showed a clonal relation between 2.3% of the isolates at ward level, 1.0% at institution level and 0.5% between institutions [29]. This finding suggests that in Dutch hospitals the transmission rate of ESBL-E between patients is low, which was also found in Swiss hospitals [30, 31]. Our findings, however, indicate that ESBL-E transmission within LTFCs might be higher.

Our study has some limitations. More than half of the initially selected LTCFs refused to participate, mainly because of time constraints. The LTCFs that did participate endorsed the importance of a point prevalence survey, and of infection control. This selection bias may have resulted in an underestimation of the MDRO prevalence.

Due to the low participation rate of residents within participating LTCFs (<20% in some LTCFs), it is not possible to make robust statements concerning transmission. Furthermore, in our study we could not associate current carriage of ESBL with known risk factors described in literature [32], except for being diagnosed with a MDRO in the preceding year. This could be due to the relative small sample size.

In conclusion, our data show that the carriage rate of ESBL-E in Amsterdam is significantly higher in LTCFs than in the general population, and varies considerably between LTCFs. The prevalence of MRSA and VRE, on the contrary, is low. No MCR-1 colistin-resistance was detected in the MDRGN isolates. Resistance due to the expansion of CTX-M ESBLs, in particular CTX-M-15, is emerging in LTCFs in Amsterdam. About half of multidrug-resistant *E*. *coli* appear to be related to the international clonal complex ST131. The majority of ESBL-E carriers are undetected in LTCFs in Amsterdam and current infection control practices do not prevent transmission. Both improvement of basic hygiene, and funding for laboratory screening, should allow LTCFs in Amsterdam to develop standards of care to prevent transmission of ESBL-E.

## Supporting information

S1 Table Primer sequences. (DOCX)

S1 File Supplementary methods. (DOCX)
