# Supplementary material for: High prevalence of multidrug resistant Enterobacteriaceae among residents of long term care facilities in Amsterdam, the Netherlands
Source: PLoS One. 2019 Sep 12;14(9):e0222200. doi: 10.1371/journal.pone.0222200 (PMC6742385; doi:10.1371/journal.pone.0222200)
Supplement: S1 Table — (DOCX) [file pone.0222200.s001.docx]

**S1 Table. Primer sequences**

| **Oligonucleotide** | **Sequence 5’-3’** | **Reference^*^** |
| --- | --- | --- |
| SHV-F1 | CTTTACTCGCCTTTATCG | 1. |
| SHV-R1 | TTAGCGTTGCCAGTGCTC | 2. |
| SHV-F2 | ACTGCCTTTTTGCGCGAGAT | 1. |
| SHV-R2 | CAGTTCCGTTTCCCAGCCGT | 1. |
| CTX-M-1F | ATGGTTAAAAAATCACTGCG | 3. (CTX-M-10-1F) |
| CTX-M-1R | CCGTTTCCGCTATTACAAAC | 4. (preCTX-M) |
| CTX-M-9F | TGGTGACAAAGAGAGTGCAACG | 3. (CTX-M-9-1F) |
| CTX-M-9R | TCCTTCAACTCAGCAAAAGT | 5. (CTX-M-9-AS) |
| TEM-F2 | TAACCATGAGTGATAACACT | 1. |
| TEM-R2 | CCGATCGTTGTCAGAAGTAA | 1. |

* Original primer names between brackets

1. Al Naiemi N, Duim B, Savelkoul PHM, Spanjaard L, De Jonge E, Bart A, Vandenbroucke-Grauls CM, de Jong MD. Widespread transfer of resistance genes between bacterial species in an intensive care unit: implications for hospital epidemiology. J. Clin. Microbiol. 2005; 43: 4862-4864.

2. Oliver A, Weigel LM, Rasheed JK, McGowan JE, Raney P, Tenover FC. Mechanism of decreased susceptibility to Cefpodoxime in Escherichia coli. Antimicrob. Agents Chemother. 2002; 46:3829-3836.

3. Paauw A, Fluit AC, Verhoef J, Leverstein-van Hall MA. Enterobacter cloacae outbreak and emergence of quinolone resistance gene in Dutch hospital. Emerg. Infect. Dis. 2006; 12:807-812.

4. Dhanji H, Patel R, Wall, R, Doumith M, Patel B, Hope R, Livermore DM, Woodford N. Variation in the genetic environment of blaCTX-M-15 in Escherichia coli from faeces of travelers returning to the United Kingdom. J. Antimicrob. Chemother. 2011; 66:1005-1012.

5. Kim J, Lim, Y-M, Rheem I, Lee Y, Lee J-C. Seol S-Y, Lee Y-C, Cho D-T. CTX-M and SHV-12 B-lactamases are the most common extended-spectrum enzymes in clinical isolates of Escherichia coli and Klebsiella pneumoniae collected from 3 university hospitals within Korea. FEMS Microbiol. Lett. 2005; 245:93-98.
