# Supplementary material for: High prevalence of multidrug resistant Enterobacteriaceae among residents of long term care facilities in Amsterdam, the Netherlands
Source: PLoS One. 2019 Sep 12;14(9):e0222200. doi: 10.1371/journal.pone.0222200 (PMC6742385; doi:10.1371/journal.pone.0222200)
Supplement: S1 File — (DOCX) [file pone.0222200.s002.docx]

## S1 File. Supplementary methods

## Laboratory detection

After overnight incubation (37°C), nasal swabs (Copan eMRSA™, Brescia, Italy) were cultured on chromID™ MRSA agar (bioMerieux, Marcy l’Etoile, France). Feces (COPAN FecalSwab™, Brescia, Italy) was cultured on chromID™ MRSA agar after overnight incubation in nutrient broth no.2 + 6% NaCl (Media Products, Groningen, the Netherlands). Feces was additionally screened for 1) multidrug-resistant Gram-negative organisms using overnight incubation of an amoxicillin (16mg/L) containing BHI broth (Media Products) subcultured to MacConkey agar plates (Media Products) with cefotaxime (5ug) and ceftazidim (10 ug) neo-sensitabs (Rosco Diagnostica, Taastrup, Denmark) and MacConkey agar plates containing 16 ug/L gentamicin with a ciprofloxacin neo-sensitab (10ug) and 2) vancomycin-resistant enterococci (VRE) using overnight incubation of an antibiotic free Enterococcosel^TM^ enrichment broth (Becton Dickinson, Utrecht, Netherlands) and chromID™ VRE (bioMerieux) agar plates. Identification and antimicrobial susceptibility testing (N200 card) of isolates was performed by standard methods using the Vitek2 instrument (bioMérieux) and phenotypic confirmation of ESBL by E-test in accordance with EUCAST (1) and Dutch national guidelines (2). Confirmation and genotyping of MRSA and CPE was performed by the Dutch reference laboratory at the National Institute for Public Health and the Environment (RIVM).

## Amplified fragment length polymorphism (AFLP)

AFLP was performed mostly as described (3). Briefly, bacterial cells were lysed with Tris-EDTA-buffer (10 mM Tris-HCL, 1 mM EDTA). The lysate was centrifuged, and the supernatant was used for AFLP. The restriction/ligation reaction mixtures consisted of approximately 10 ng DNA, 1x T4 DNA ligase buffer, 0.05 M NaCl, 1 µg BSA, 2 pmol of the EcoRI adapter, 20 pmol of the MseI adapter (Eurogentec, Maastricht, the Netherlands), 160 U of T4 DNA ligase, 2 U of EcoRI and 2 U of MseI. All enzymes were purchased from New England Biolabs (Leiden, Netherlands). After incubation at 37ºC for 1 h, the mixtures were diluted 1:20 in water. 5 µl of the mixture was added to 5 µl of PCR mixture, which consisted of 1x PCR buffer (Sphaero Q, Gorinchem, The Netherlands), 2,5 mM MgCl, 350 µM dNTPs (Promega, Leiden, Netherlands), 1 U Super Taq Plus polymerase (Sphaero Q), and 20 ng of Eco-A primer and 60 ng of Mse-C primer (4). The Eco-A primer was fluorescently labelled with carboxyfluorescein (Eurogentec). Amplification was carried out under the following conditions: 2 min at 72ºC, followed by 12 cycles of 30 s at 94ºC, 30 s starting at 65ºC and gradually reduced by 0.7 ºC per cycle, and 1 min at 72ºC, and then 23 cycles of 30 s at 94ºC, 30 s at 56ºC and 1 min at 72ºC and ended by a single extension at 72ºC for 10 min. 2.5 µl of each PCR product was added to 22 µl Hi-Di formamide and 0.5 µl GeneScan-600 LIZ size standard (Applied Biosystems, Bleiswijk, Netherlands). Fragments were separated on an ABI Prism 3130 sequencer (Applied Biosystems). Data were analysed with the GENESCAN analysis software (Applied Biosystems) and BioNumerics software package, version 6.6 (Applied Maths, Sint-Martens-Latem, Belgium). Similarity coefficients were calculated with Pearson correlation and dendrograms were obtained by the unweighted pair group method using arithmetic averages (UPGMA) clustering. The analysis was performed for fragments with lengths between 60 and 600 bp. Isolates were considered indistinguishable (representing a cluster) when the band patterns were identical.

## Phylogenetic typing

A selection of *E. coli* strains was subjected by phylogroup-defining PCR (5). Group B2 *E. coli* underwent O25:ST131-specific PCR (6).

## Detection of resistance genes

The presence of CTX-M, SHV and TEM ESBL genes was confirmed by High Resolution or SYBR Green melting curve analysis PCR of cell lysates from all phenotypically confirmed ESBL-positive strains. (CTX-M primer sequences in Mulvey et al. 2003 (7), TEM and SHV primer sequences in Agerso et al. 2012 (8)). The PCR contained Precision Melt Supermix (CTX-M PCR) or SYBR Green Supermix (SHV and TEM PCR) buffers (Bio-Rad, Veenendaal, Netherlands), 150 nM of each primer, and bacterial lysate. Amplification was carried out in a LightCycler 480 instrument (Roche, Almere, The Netherlands) under the following conditions: 10 min at 95ºC, followed by 35 cycles of 30 s at 95ºC, 30 s at 55 ºC and 45 s at 72ºC. Hereafter 1 min at 95ºC and 1 min at 40 ºC. A melting curve was recorded by heating between 65ºC and 95º with a ramp rate of 0.02 ºC/s. Melting curves were converted to melting peaks by the LightCycler software. Distinct peaks were registered for CTX-M groups 1, 2, 8 and 9.

Fecal samples were subjected to MCR-1 PCR by the Leiden University Medical Centre according to methods described previously by Nijhuis et al. (9) and Terveer et al. (10).

**Molecular typing of ESBL genes**

The SHV, TEM and CTX-M genes were typed by sequencing. The TEM products obtained above were directly used for sequencing with the PCR primers and primers TEM-F2 and TEM-R2. The SHV genes were amplified with primers SHV-F1 and SHV-R1 and sequenced with amplification primers and primers SHV-F2 and SHV-R2. CTX-M groups 1 and 9 were amplified with primers CTX-M-1F and CTX-M-1R, and CTX-M-9F and CTX-M-9R, respectively. Sequencing was performed with amplification primers and the PCR primers used for detection of resistance genes. Amplification protocol was as above (detection of resistance genes). Primer sequences are listed in Supplementary Table 1. All PCR products were purified with the QIAquick PCR purification kit according to the instructions of the manufacturer (Qiagen, Venlo, Netherlands). Sanger sequencing was performed with the BigDye Terinator v3.1 Cycle Sequencing Kit (Applied Biosystems, Bleiswijk, Netherlands) with 2 pmol/ µl primer. Sequence conditions were 1 min at 96ºC, followed by 25 cycles of 10 s at 96ºC, 5 s at 55 ºC and 4 min at 60ºC, ending at 4ºC. Sequence products were precipitated with isopropanol and dissolved in formamide, after which fragments were separated on an ABI Prism 3130 sequencer (Applied Biosystems). Sequences were analysed with the CodonCode Aligner software (CodonCode Corporation, Centerville, USA). Consensus sequences were uploaded at The Comprehensive Antibiotic Resistance Database BLAST service for typing (Jia et al., at [http://arpcard.mcmaster.ca](http://arpcard.mcmaster.ca/)). (11)

**References**

1. The European Committee on Antimicrobial Susceptibility Testing - EUCAST. Breakpoint Tables for Interpretation of MICs and Zone Diameters. [Internet]. Available from: <http://www.eucast.org/clinical_breakpoints/>.

2. NVMM Guideline Laboratory detection of highly resistant microorganisms, version 2.0, 2012. <http://www.nvmm.nl/media/1051/2012_hrmo_mrsa_esbl.pdf>.

3. Mohammadi T, Reesink HW, Pietersz RN, Vandenbroucke-Grauls CM, Savelkoul PH. Amplified-fragment length polymorphism analysis of Propionibacterium isolates implicated in contamination of blood products. Br J Haematol. 2005;131(3):403-9.

4. Savelkoul PH, Aarts HJ, de HJ, Dijkshoorn L, Duim B, Otsen M, et al. Amplified-fragment length polymorphism analysis: the state of an art. J Clin Microbiol. 1999;37(10):3083-91.

5. Doumith M, Day MJ, Hope R, Wain J, Woodford N. Improved multiplex PCR strategy for rapid assignment of the four major Escherichia coli phylogenetic groups. J Clin Microbiol. 2012;50(9):3108-10.

6. Dhanji H, Doumith M, Clermont O, Denamur E, Hope R, Livermore DM, et al. Real-time PCR for detection of the O25b-ST131 clone of Escherichia coli and its CTX-M-15-like extended-spectrum beta-lactamases. Int J Antimicrob Agents. 2010;36(4):355-8.

7. Mulvey MR, Soule G, Boyd D, Demczuk W, Ahmed R, Multi-provincial Salmonella Typhimurium Case Control Study G. Characterization of the first extended-spectrum beta-lactamase-producing Salmonella isolate identified in Canada. J Clin Microbiol. 2003;41(1):460-2.

8. Agerso Y, Aarestrup FM, Pedersen K, Seyfarth AM, Struve T, Hasman H. Prevalence of extended-spectrum cephalosporinase (ESC)-producing Escherichia coli in Danish slaughter pigs and retail meat identified by selective enrichment and association with cephalosporin usage. J Antimicrob Chemother. 2012;67(3):582-8.

9. Nijhuis RH, Veldman KT, Schelfaut J, Van Essen-Zandbergen A, Wessels E, Claas EC, et al. Detection of the plasmid-mediated colistin-resistance gene mcr-1 in clinical isolates and stool specimens obtained from hospitalized patients using a newly developed real-time PCR assay. J Antimicrob Chemother. 2016;71(8):2344-6.

10. Terveer EM, Nijhuis RHT, Crobach MJT, Knetsch CW, Veldkamp KE, Gooskens J, et al. Prevalence of colistin resistance gene (mcr-1) containing Enterobacteriaceae in feces of patients attending a tertiary care hospital and detection of a mcr-1 containing, colistin susceptible E. coli. PLoS One. 2017;12(6):e0178598.

11. Jia B, Raphenya AR, Alcock B, Waglechner N, Guo P, Tsang KK, et al. CARD 2017: expansion and model-centric curation of the comprehensive antibiotic resistance database. Nucleic Acids Res. 2017;45(D1):D566-D73.
